# Excision of the Uvula and Tonsil, for the Cure of Stammering

**Published:** 1841-10

**Authors:** 


*Excision of the Uvula and Tonsil, for the cure of Stammering.*—A Mr. Poett has been investigating the merits of Mr. Yearsley's operation, which, like a great many other novel remedies, will not bear a close inspection. A Mr. Butler, one of the earliest cases he operated on, and a very formidable one, had, as soon as his throat healed, a return of his disease. Two boys, reported to have stuttered and been cured, proved on inquiry, the one never to have done so, the other to be as bad as ever. Case number five was no better, and two could not be found at the address given in the work. Several others owned that at first they felt some relief, but when excited, or agitated, stuttered as badly as before. When once the influence of the operation on the nervous system has subsided, the infirmity will probably re-appear, and though the simple and easy operation may be tried with a faint prospect of benefit, no reliance can be placed on it, and it will probably share the fate of all over-rated novelties, and fall into deserved oblivion. Mr. Yearsley has greatly deceived himself or others. This operation is not "what it was cracked up to be." Shall we be permitted, descending from "grave to gay," to append a little anecdote as somewhat "germane to the matter," and *the times?* A gentleman who stuttered badly, put himself under the care of a celebrated curer of the infirmity, and derived, as he thought, much benefit from his advice. Meeting one day, a friend who stuttered as badly as he had done, he very kindly said to him, "W-w-w-why, m m-m-my d-d-dear S-s-sir, d-d-d-don't you g-g-g-go to the m-m-m-man that cu-cu-cu--cured me?" Mr. Poett's paper is in the Lancet for June 12, 1841.

A Reviewer, in the last number of the British and Foreign, states that he too has taken pains to look up some of Mr. Yearsley's *cured* cases, and from personal examination he condemns, as every man of sense and honor must, this absurd operation.—*N. Y. Med. Gaz.*

